# Evolutionary context for the association of γ-globin, serum uric acid, and hypertension in African Americans

**DOI:** 10.1186/s12881-015-0249-z

**Published:** 2015-11-05

**Authors:** Daniel Shriner, Chutima Kumkhaek, Ayo P. Doumatey, Guanjie Chen, Amy R. Bentley, Bashira A. Charles, Jie Zhou, Adebowale Adeyemo, Griffin P. Rodgers, Charles N. Rotimi

**Affiliations:** Center for Research on Genomics and Global Health, National Human Genome Research Institute, Building 12A/Rm 4047, 12 South Dr., Bethesda, MD 20892 USA; Molecular and Clinical Hematology Branch, National Heart, Lung, and Blood Institute, Bethesda, MD USA

**Keywords:** African American, Ancestry, Gamma-globin, Health disparity, Hypertension, Malaria, Uric acid

## Abstract

**Background:**

Hyperuricemia and associated cardio-metabolic disorders are more prevalent in African Americans than in European Americans. We used genome-wide admixture mapping and association testing to identify loci with ancestry effects on serum uric acid levels.

**Methods:**

We analyzed 1,976 African Americans from Washington, D.C, including 1,322 individuals from 328 pedigrees and 654 unrelated individuals, enrolled in the Howard University Family Study. We performed admixture mapping and genome-wide association testing using ~800 k autosomal single-nucleotide polymorphisms (SNPs). We performed fine mapping by dense genotyping. We assessed functionality by a combination of bioinformatic annotation, reporter gene assays, and gel shift experiments. We also analyzed 12,641 individuals enrolled in the National Health and Nutrition Examination Survey.

**Results:**

We detected a genome-wide significant locus on chromosome 11p15.4 at which serum uric acid levels increased with increasing African ancestry, independent of kidney function. Fine-mapping identified two independent signals in the β-globin locus. The ancestral allele at SNP rs2855126, located upstream of the hemoglobin, gamma A gene *HBG1*, was associated with increased serum uric acid levels and higher expression of a reporter gene relative to the derived allele. Hyperuricemia was associated with increased risk of hypertension in 3,767 African Americans (Odds Ratio = 2.48, *p* = 2.71 × 10^− 19^).

**Conclusions:**

Given that increased expression of γ-globin leads to increased levels of fetal hemoglobin which confers protection against malaria, we hypothesize that evolution in Africa of protection against malaria may have occurred at the cost of increased serum uric acid levels, contributing to the high rates of hyperuricemia and associated cardio-metabolic disorders observed in African Americans.

**Electronic supplementary material:**

The online version of this article (doi:10.1186/s12881-015-0249-z) contains supplementary material, which is available to authorized users.

## Background

Uric acid is the most abundant anti-oxidant in human plasma [1]. The primary endogenous source of uric acid is degradation of extruded nuclei from normoblasts during erythropoiesis [2]. An elevated level of serum uric acid, hyperuricemia, is a risk factor for gout, and is implicated in both cardiovascular disease and metabolic disease [3]. In particular, hyperuricemia is associated with an increased risk of hypertension that occurs at serum uric acid concentrations lower than the supersaturation value, indicating a risk for hypertension independent of the development of gout [4]. Heritability estimates of serum uric acid range from 35 % to 40 % [5–7], indicating the presence of genetic variants influencing serum uric acid levels. A recent genome-wide association study (GWAS) identified several loci influencing serum uric acid levels in both European Americans and African Americans [8]. Furthermore, meta-analysis of GWAS in individuals of European ancestry identified 26 loci accounting for 7.0 % of the phenotypic variance in serum uric acid levels [9].

The prevalence of hyperuricemia in the US is currently ~21 % and has been increasing over the past few decades [10]. The estimated prevalence of hyperuricemia is 25.7 % in African Americans, compared to 22.1 % in European Americans [10]. Compared to European Americans, a higher prevalence in African Americans has been observed for cardiovascular and metabolic diseases associated with hyperuricemia such as hypertension [11], obesity [12], and type 2 diabetes [13]. It is unknown how much of the higher prevalence of hyperuricemia in African Americans is due to genetic *vs*. environmental risk factors such as diet. Here, we used joint admixture mapping and association testing to identify genetic variants associated with serum uric acid levels in African Americans. This technique is designed to discover genetic variants differentially contributing to variance in serum uric acid levels between the West African and European ancestries of admixed African Americans. We then performed bioinformatic annotation, luciferase reporter gene assays, and gel shift experiments to assess associated genetic variants for functionality.

## Methods

### Ethics statement

Ethics approval for the Howard University Family Study was obtained from the Howard University Institutional Review Board. Written informed consent was obtained from each participant. All clinical investigation was conducted according to the principles expressed in the Declaration of Helsinki.

### Study samples

The Howard University Family Study (HUFS) is a population-based genetic epidemiology study of African Americans in Washington, D.C. Enrollment occurred in two phases, the first consisting of 1,322 individuals from 328 families and the second consisting of 654 unrelated individuals. Study participants were not ascertained for any phenotype. Using the Affymetrix Genome-Wide Human SNP Array 6.0 (Affymetrix, Santa Clara, California) and established quality control filters [14], we obtained genome-wide genotypes for 808,465 autosomal single nucleotide polymorphisms (SNPs) for all 1,976 individuals.

The National Health and Nutrition Examination Survey (NHANES) is a nationally representative, population-based epidemiological study of health and nutritional status (http://www.cdc.gov/nchs/nhanes/about_nhanes.htm). This study included 2,841 Mexican Americans, 6,907 non-Hispanic Whites, and 2,893 non-Hispanic Blacks.

### Local ancestry

We estimated local ancestry, *i.e.*, 0, 1, or 2 chromosomes of African ancestry, for 797,831 unique autosomal SNPs using LAMPANC version 2.3 [15] and reference allele frequencies for the HapMap Phase II + III CEU (Utah residents with northern and western European ancestry) and YRI (Yoruba in Ibadan, Nigeria) samples (http://hapmap.ncbi.nlm.nih.gov/downloads/frequencies/2010-08_phaseII+III/). Genome-wide, the average proportion of African ancestry, also known as the individual admixture proportion or global ancestry, was 0.799.

### Heritability of serum uric acid levels

The heritability of serum uric acid levels was estimated based on 1,314 individuals in 328 pedigrees from HUFS using SOLAR version 4.1.2 [16].

### Phenotypes

Serum uric acid levels were assessed using the COBAS INTEGRA UA2 test (Roche Diagnostics, Indianapolis, Indiana). Serum uric acid values were Box-Cox transformed due to non-normality. Hyperuricemia was defined as serum uric acid > 7.0 mg/dL in males and > 6.0 mg/dL in females. Serum creatinine levels were estimated from fasting blood samples using the COBAS INTEGRA CREJ2 test (Roche Diagnostics). The estimated glomerular filtration rate (eGFR) was calculated using the Modification of Diet in Renal Disease Study equation: eGFR = 186 × (serum creatinine)^-1.154^ × age^-0.203^ (×0.742 if female) (×1.210 if Black), with serum creatinine measured in mg/dL and eGFR measured in mL/min/1.73 m^2^ [17].

### Joint ancestry and association testing

Using R software [18], we performed joint ancestry and association testing as described previously [19]. Briefly, we first performed ancestry testing, also known as admixture mapping, using linear regression of serum uric acid level as a function of local ancestry, adjusted for age, sex, and individual admixture proportion. Based on autocorrelation of local ancestry, the empirical genome-wide testing burden of admixture mapping was 370.7, leading to a genome-wide significance level of $\frac{0.05}{370.7}=1.35\times {10}^{-4}$. Given a significance level of 1.35 × 10^− 4^ and an average of 79.9 % African ancestry, we estimated that our sample provided 80 % power to detect a locus explaining 2.13 % of the phenotypic variance. We performed association testing using linear regression of serum uric acid level as a function of genotype stratified by local ancestry and adjusted for age, sex, and individual admixture proportion. We combined the association results across strata using inverse variance-weighted fixed effects meta-analysis. Based on autocorrelation of genotype, the empirical genome-wide testing burden of association testing was 345470.4, leading to a genome-wide significance level of $\frac{0.05}{345470.4}=1.45\times {10}^{-7}$. We then combined the results from admixture mapping and association testing, using the results from admixture mapping as prior probabilities for association testing in the Bayesian framework.

### Fine-mapping

Based on the 1000 Genomes sequence data for the YRI sample [20], we identified 152 tag SNPs for the β-globin locus that provided 90 % coverage at *r*^2^ ≥ 0.8 of SNPs with a minor allele frequency ≥1 % and 79 % coverage of all SNPs [21]. Of the 152 tag SNPS, primer design was successful for 145 and genotyping was successful for 132. Genotyping was performed using the iPlex Gold assay on the MassARRAY platform (Sequenom, Inc., San Diego, California).

### Construction of expression vectors

DNA samples harboring each SNP were amplified by polymerase chain reaction (PCR) using the AccuPrime™ Taq DNA Polymerase System (Life Technologies, Carlsbad, California). Primers used to amplify DNA fragments are shown in Additional file 1. PCR products were first subcloned into the pCR®2.1-TOPO vector (Life Technologies) and modified by site-directed mutagenesis using the QuikChange Lightning Site-Directed Mutagenesis Kit (Agilent Technologies, Santa Clara, California) to generate the other allele. To confirm sequence identity, positive clones were fully sequenced in both directions (Eurofins MWG Operon, Huntsville, Alabama). For each SNP, an allele-positive clone was subcloned into the selected restriction enzyme-digested pGL3-Basic luciferase vector (Promega, Madison, Wisconsin) using the Quick Ligation™ kit (New England BioLabs, Ipswich, Massachusetts) with subsequent transformation into JM109 competent cells (Promega). All clones were also sequence-verified (Eurofins MWG Operon).

### Cell culture and luciferase activity assay

K562 (an erythroleukemic cell line persistently expressing fetal hemoglobin) and 293 T (a transformed human embryonic kidney cell line) cells were obtained from the American Type Culture Collection (ATCC, Manassas, VA, USA). K562 cells were cultured in Roswell Park Memorial Institute (RPMI) 1640 medium (Life Technologies). 293 T cells were cultured in Dulbecco’s modified Eagle’s medium (Life Technologies). All media contained 10 % fetal bovine serum (Life Technologies) and 1 % penicillin/streptomycin (Life Technologies). For dual luciferase reporter gene assays, cells were grown in 24-well plates and co-transfected with 800 ng of firefly luciferase vector constructs containing the SNP fragments and 80 ng of TK *Renilla* luciferase vector (pRL-TK vector, Promega) using Lipofectamine 2000 (Life Technologies). Forty-eight hours after transfection, cells were harvested and luciferase activity was measured using the Dual-Luciferase® Reporter Assay System (Promega) according to the manufacturer’s protocols. Luciferase activity was normalized using the ratio between the firefly luciferase activity and the TK *Renilla* luciferase activity.

### Electrophoretic mobility shift assay (EMSA)

Non-radioactive EMSAs were performed using a LightShift Chemiluminescent EMSA kit (Thermo Scientific, Rockford, IL, USA) and two biotin-labeled synthetic oligonucleotides containing either ancestral or derived alleles (Eurofins MWG Operon). Non-biotin-labeled synthetic oligonucleotides with the same sequences were used as competitors. Nuclear extracts from K562 cells were prepared using NE-PER nuclear and cytoplasmic extraction reagents (Thermo Scientific), incubated with 20 fmol of biotin-labeled synthetic oligonucleotides for 20 minutes at room temperature and electrophoresed on 6 % Novex DNA retardation gels (Life Technologies). In competition reactions, nuclear extracts were incubated with 4 pmol of unlabeled synthetic oligonucleotides. Epstein-Barr nuclear antigen (EBNA) extract and control DNA were used as a positive control. In super-shift experiments, the extracts were pre-incubated with antibodies (Santa Cruz Biotechnology, Dallas, TX, USA) for 60 min on ice. Chemiluminescent signals were developed according to the manufacturer’s instructions.

## Results

### Genetic mapping of serum uric acid in African Americans

**Fig. 1:**
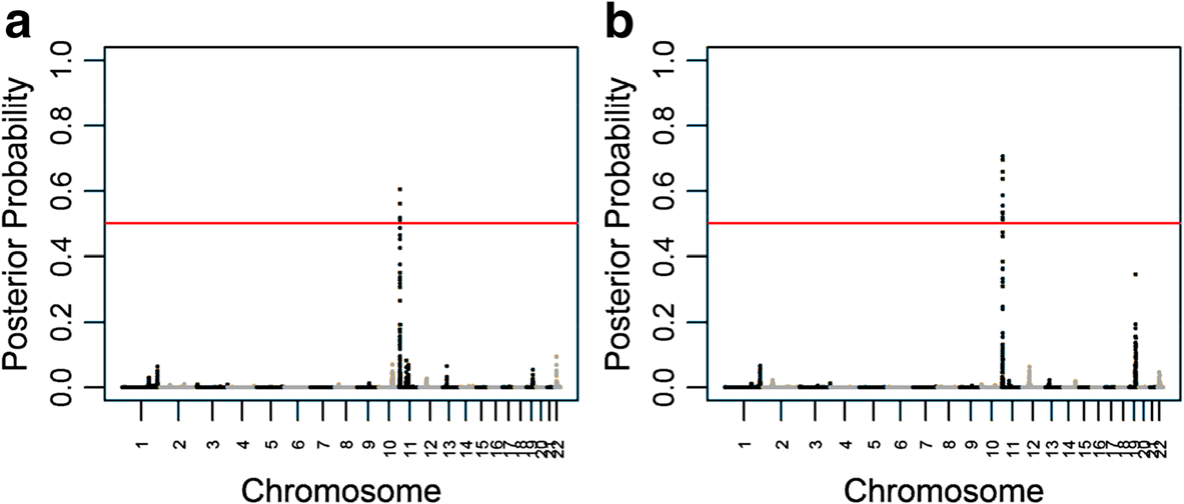
Admixture mapping for serum uric acid levels. Red lines indicate the genome-wide significance level. **a** Unadjusted and **b** adjusted for the estimated glomerular filtration rate

The heritability of serum uric acid levels was estimated to be 35.4 % with a standard error of 6.6 %, providing strong evidence for an additive genetic component. We next analyzed serum uric acid levels in 1,007 unrelated African Americans who were at least 20 years old. The sample comprised 414 males and 593 females with an average age of 48.3 years (standard deviation [SD] = 13.2 years) and an average of 79.9 % African ancestry (SD = 11.6 %). Admixture mapping yielded one genome-wide significant peak (likelihood of odds [LOD] score = 3.20, *p* = 1.24 × 10^− 4^) at chromosome 11p15.4 (Fig. 1a). The 1-LOD interval, or approximately the 95 % confidence interval, extended from 4,791,111 bp to 5,665,225 bp (GRCh37 coordinates). At this locus, serum uric acid levels increased with increased African ancestry. This locus explained 1.27 % of the variance in serum uric acid levels.

Increased serum uric acid levels could reflect increased production or decreased renal excretion. To distinguish between these two possibilities, we performed linear regression of serum uric acid as a function of local ancestry adjusted for age, sex, and individual admixture proportion, with an additional adjustment for eGFR. Adjustment for eGFR did not substantially alter the effect of African ancestry on serum uric acid levels at 11p15.4 (1.27 % variance explained, *p* = 1.24 × 10^− 4^ without adjustment compared to 1.20 % variance explained, *p* = 8.31 × 10^− 5^ with adjustment, Fig. 1b), indicating that the genetic association at 11p15.4 was essentially unaffected by eGFR. Therefore, increased serum uric acid levels were more likely due to increased production of uric acid rather than decreased renal excretion.

**Fig. 2:**
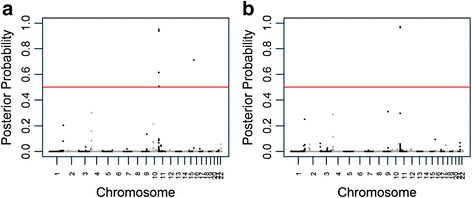
Joint ancestry and association testing for serum uric acid levels. Red lines indicate the genome-wide significance level. **a** Unadjusted and **b** adjusted for the estimated glomerular filtration rate

To fine-map this locus, we performed association testing and combined the results with the prior admixture mapping. After adjusting for eGFR in addition to age, sex, and individual admixture proportion, the signal resolved to two SNPs: rs2855126, with a posterior probability of a joint ancestry and association effect of 0.965; and rs2855123, with a posterior probability of a joint ancestry and association effect of 0.971 (Fig. 2). Both SNPs map to the β-globin locus. rs2855126 is located at 5,273,147 bp, 2,060 bp upstream of the hemoglobin, gamma A gene *HBG1*. rs2855123 is located at 5,277,078 bp, 1,067 bp upstream of the hemoglobin, gamma G gene *HBG2*. These two SNPs are strongly correlated in the 1000 Genomes ASW (Americans of African ancestry in southwest USA, *r*^2^ = 1), CEU (*r*^2^ = 0.973), and YRI (*r*^2^ = 1) samples, as well as our HUFS data set (*r*^2^ = 0.988). At both SNPs, the derived allele is associated with lower serum uric acid levels and is present at lower frequencies in the YRI sample than in the CEU sample (Table 1), consistent with the results of admixture mapping that demonstrated increased serum uric acid levels with increased African ancestry.

**Table 1:** Association of SNPs in the β-globin locus with serum uric acid levels.

	rs2855126	rs2855123
Chromosome	11	11
Position, bp	5,273,147	5,277,078
Coded Allele	G	T
Other Allele	C	A
Ancestral Allele	C	A
Freq_HUFS_	0.247	0.246
Freq_ASW_	0.353	0.353
Freq_CEU_	0.412	0.406
Freq_YRI_	0.176	0.176
*P*_Allele_^a^	6.80 × 10^−7^	1.29 × 10^−6^
Beta_Meta_^b^	−0.125	−0.127
SE_Meta_^c^	0.0338	0.0341
*P*_Meta_^d^	2.27 × 10^−4^	1.99 × 10^−4^
*P*_Het_^e^	0.818	0.739
I^2f^	0	0

HUFS indicates the Howard University Family Study; ASW, Americans of African Ancestry in SW USA; CEU, Utah Residents (CEPH) with Northern and Western European ancestry; and YRI, Yoruba in Ibadan, Nigeria.

^a^ *P*-value from the test of allele frequencies from the stratum of 0 African chromosomes *vs*. 2 African chromosomes.

^b^Inverse variance-weighted effect size across the three ancestry strata. For rs2855126, the effect size corresponds to 0.357 mg/dL per allele.

^c^Inverse variance-weighted standard error across the three ancestry strata.

^d^Association *p*-value for the effect size and standard error combined across the three ancestry strata.

^e^ *P*-value from Cochran’s Q test for heterogeneity in effect size across ancestry strata.

^f^The percentage of heterogeneity in effect size estimates due to between-strata heterogeneity.

**Fig. 3:**
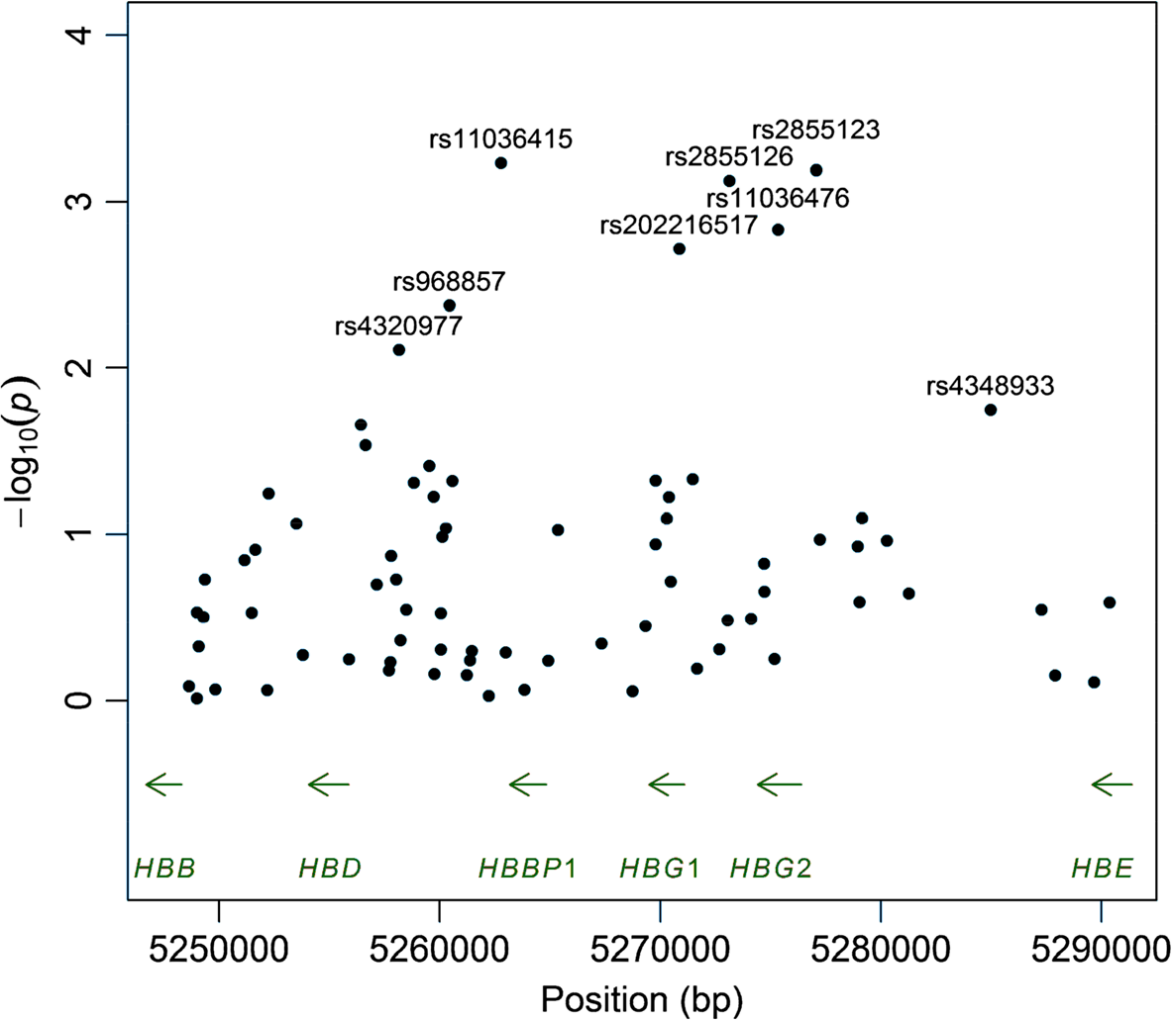
Association testing for serum uric acid levels based on dense genotyping of the β-globin locus.

To further interrogate the β-globin locus, we performed genotyping for 152 tag SNPs. With these additional data, we identified one SNP, rs11036415, more strongly associated with serum uric acid levels than either rs2855126 or rs2855123 (Fig. 3). rs11036415 is located 403 bp downstream of hemoglobin, beta pseudogene 1 *HBBP1* and 6.9 kb upstream of the hemoglobin, delta gene *HBD*. Conditioning on rs2855126 eliminated the association at rs2855123 (*p* = 0.788) but not the association at rs11036415 (*p* = 0.00427). Similarly, conditioning on rs11036415 did not eliminate the association at rs2855126 (*p* = 0.00457) or rs2855123 (*p* = 0.00467). Therefore, the associations at these three SNPs reflect two distinct signals.

Hyperuricemia is common in individuals with sickle cell disease [22], raising the possibility that the variant rs334 in the hemoglobin, beta gene *HBB* that leads to hemoglobin S in sickle-cell disease was driving the association we observed between the β-globin locus and serum uric acid levels. Unfortunately, rs334 could not be genotyped using our approach. However, based on the 1000 Genomes Project ASW sequence data, rs334 is not correlated with rs2855123 (*r*^2^ = 0.030), rs2855126 (*r*^2^ = 0.030), or rs11036415 (*r*^2^ = 0.050), suggesting an effect independent of rs334 and *HBB*. Similarly, the −158 C → T variant in the *HBG2* promoter (rs7482144), associated with hereditary persistence of fetal hemoglobin [23], is not correlated with rs2855123 (*r*^2^ = 0.020), rs2855126 (*r*^2^ = 0.020), or rs11036415 (*r*^2^ = 0.010) in the 1000 Genomes Project ASW sequence data.

### Bioinformatic annotation

We next performed bioinformatic annotation for the top associated SNPs at the β-globin locus. Using rs2855123, rs2855126, and rs11036415, we queried HaploReg v2 [24] for variants with *r*^2^ ≥ 0.8 in the sequence data from the 1000 Genomes African samples. Three SNPs (rs2855126, rs2855125, and rs11036496) were annotated as binding proteins based on ChIP-seq experiments (Additional file 2). Based on the dense genotyping and annotation, we selected rs2855125 (protein binding, DNase I hypersensitive site, and promoter histone marks), rs2855126 (protein binding and enhancer histone marks), rs11036415 (top association and enhancer histone marks), and rs11036496 (protein binding, DNase I hypersensitive site, and enhancer histone marks), as well as rs4320977 and rs4348933 (chosen based on the possibility that both of these variants are associated and that the peak of association maps to somewhere in between these two SNPs) for follow-up functional analysis.

SNPs rs4348933 and rs11036496 are located 9.0 kb and 4.0 kb upstream of *HBG2*, respectively. SNPs rs2855125 and rs2855126 are located 2.6 kb and 2.1 kb upstream of *HBG1*, respectively. SNPS rs11036415 and rs4320977 are located 6.9 kb and 2.3 upstream of *HBD*, respectively. We found no promoter regions in DNA sequences surrounding each of the six SNPs using Promoter Inspector (Genomatix Software Inc., Ann Arbor, MI, USA). In contrast, all six SNPs mapped to transcription factor binding sites using SNP Inspector (Genomatix Software Inc.). None of the associated SNPs are protein-coding; rather, the associated SNPs are annotated with regulatory functions more consistent with enhancers than promoters.

### Reporter gene expression studies of intergenic SNPs in the β-globin cluster

**Fig. 4:**
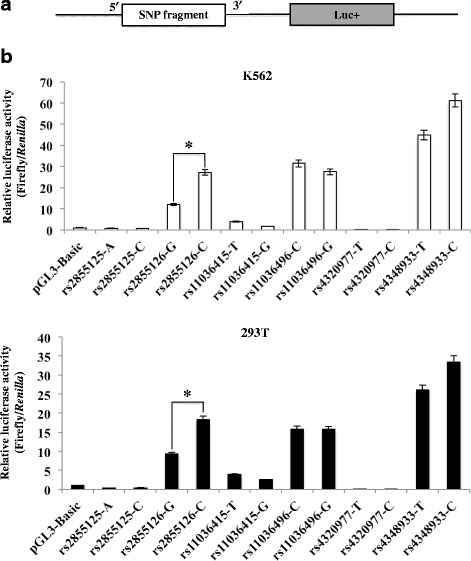
Luciferase expression results from transfected K562 and 293 T cells. **a** Schematic illustration of luciferase reporter constructs for each SNP. **b** Luciferase reporter constructs containing ancestral alleles (rs2855125-A, rs2855126-G, rs11036415-G, rs11036496-G, rs4320977-T, and rs4348933-T) or derived alleles (rs2855125-C, rs2855126-C, rs11036415-T, rs11036496-C, rs4320977-C, and rs4348933-C) were transiently transfected into K562 or 293 T cells. Cell lysates were analyzed for firefly and *Renilla* luciferase activity and the ratio of firefly/*Renilla* luciferase fluorescence was calculated. Data represent the average ± one SD from three replicates, * *p* ≤ 0.05.

To determine the potential activities of the six SNPs as enhancers or repressors of gene expression, we cloned fragments containing the six SNPs into a firefly luciferase reporter vector (one fragment for each allele, yielding 12 constructs) and co-transfected K562 or 293 T cells with both firefly and *Renilla* luciferase vectors. Expression of firefly luciferase driven by each allele-containing DNA fragment was measured by a dual luciferase reporter assay and normalized using *Renilla* luciferase expression. SNPs rs2855126, rs11036496, and rs4348933 had significantly greater expression levels of firefly luciferase than pGL3-Basic-transfected cells in both cell lines (*p* < 0.05, Fig. 4). Of these three SNPs, only rs2855126 showed differential activity by allele, with the ancestral allele C showing significantly higher luciferase activity than the derived allele G (*p* < 0.05).

### Binding of nuclear proteins to DNA sequences harboring rs2855126, rs11036496, and rs4348933

**Fig. 5:**
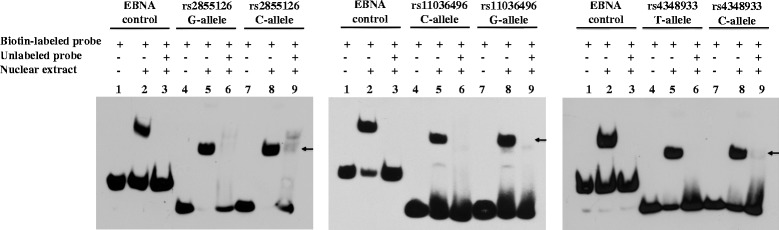
Binding of nuclear proteins from K562 cells with SNPs rs2855126, rs11036496, and rs4348933 determined by EMSA. Lanes 1, 4, and 7: in the absence of nuclear extract, biotin-labeled probe shows no retardation; lanes 2, 5, and 8: in the presence of nuclear extract, biotin-labeled probe shows retardation and gel shift due to the slower migration of protein-probe complex; and lanes 3, 6, and 9: in the presence of nuclear extract and unlabeled probe (as competitor), biotin-labeled probe shows no retardation. For both probe and nuclear extract, + indicates presence and - indicates absence.

**Fig. 6:**
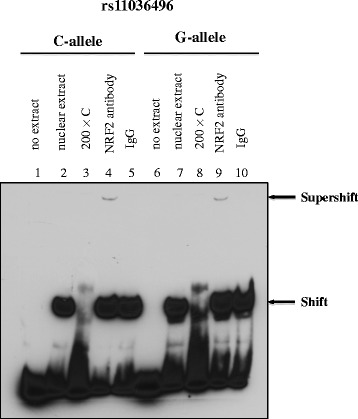
Gel supershift assay with the addition of an anti-NRF2 antibody but not with an isotype control (IgG) identifies NRF2 as a transcription factor binding with DNA sequences surrounding rs11036496. NRF2 binding was not different by allele across three individual experiments (*p* = 0.15).

Gene expression can be modulated by regulatory factor-binding sites in intronic regions [25]. To investigate the molecular mechanism underlying the transcriptional activity associated with rs2855126, rs11036496, and rs4348933, we determined whether the sequences flanking these SNPs could serve as protein-binding sites. EMSAs using nuclear extracts isolated from K562 cells demonstrated gel shifts for both alleles of all three SNPs that could be disrupted by excess unlabeled probe, indicating strong *in vitro* binding of nuclear protein to the sequences surrounding rs2855126, rs11036496, and rs4348933 (Fig. 5). We further attempted to determine which transcription factors bound to the sequences surrounding these SNPs using MatInspector (Genomatix Software Inc.) and RegulomeDB (http://www.regulomedb.org). According to these two sources, the three SNPs exhibited the potential capacity to bind with 13 transcription factors (Additional file 3). Subsequent analysis using a supershift assay demonstrated that rs11036496 was located within a binding site for NRF2 (Fig. 6), but that binding was not different by allele (*p* = 0.15). No binding was detected for any of the other 12 transcription factors.

### Biochemical and clinical characterization of hyperuricemia in nationally representative data

To better understand the biochemical and clinical nature of increased serum uric acid levels, we analyzed biochemical data in non-Hispanic Blacks enrolled in the National Health and Nutrition Examination Survey (NHANES). We observed significant association of hyperuricemia with increased levels of lactate dehydrogenase and total bilirubin (Table 2), which along with increased serum uric acid levels are markers for increased hemolysis. Hyperuricemia was also associated with increased red blood cell count, increased red cell distribution width, and hematocrit (Table 2). In contrast, hyperuricemia was not associated with hemoglobin, iron, mean cell volume, mean cell hemoglobin, or mean cell hemoglobin concentration (Table 2). Taken together, these data suggest that hyperuricemia is associated with increased hemolysis but not anemia, further suggesting a compensatory mechanism by which increased hemolysis does not lead to reduced red blood cell count. Clinically, these results are more consistent with hereditary persistence of fetal hemoglobin than β-thalassemia [23].

**Table 2:** Biochemical characterization of hyperuricemia in 2,882 NHANES non-Hispanic Blacks.

	Effect Size ^□^	*P*
LDH, U/L	7.88	2.06 × 10^−5^
BIL, mg/dL	0.0338	0.0355
RBC, 10^6^ cells/μL	0.0529	0.0234
RDW, %	0.263	3.10 × 10^−4^
HCT, %	0.346	0.0471
HGB, g/dL	0.107	0.0783
MCV, fL	−0.267	0.396
MCH, pg	−0.121	0.340
MCHC, g/dL	−0.0271	0.568
Iron, μg/dL	−1.44	0.323

LDH indicates lactate dehydrogenase; BIL, total bilirubin; RBC, red blood cell count; RDW, red cell distribution width; HCT, hematocrit; HGB, hemoglobin; MCV, mean cell volume; MCH, mean cell hemoglobin; and MCHC, mean cell hemoglobin concentration.

^□^ Effect sizes from linear regression of variable as a function of hyperuricemia case/control status, adjusted for sex and age. Hyperuricemia was defined as serum uric acid > 7.0 mg/dL in males or > 6.0 mg/dL in females.

Hyperuricemia has also been reported to be a risk factor for hypertension. In our HUFS data set, the prevalence of hypertension increased from 42.4 % in controls to 72.7 % in hyperuricemic cases. We found that hyperuricemia increased the risk of hypertension in HUFS, NHANES non-Hispanic Blacks, NHANES non-Hispanic Whites, and NHANES Mexican Americans (Table 3). Combining the HUFS and NHANES data (the estimates of risk from HUFS and NHANES non-Hispanic Blacks were not different, *p* = 0.285), we estimated a 2.48-fold increased risk (95 % confidence interval 2.03 to 3.02, *p* = 2.71 × 10^− 19^) of hypertension as a function of hyperuricemia in African Americans. We estimated a 1.85-fold increased risk (95 % confidence interval 1.62 to 2.12, *p* = 3.64 × 10^− 19^) of hypertension as a function of hyperuricemia in Mexican Americans and non-Hispanic Whites (the estimates from these two ethnicities were not different, *p* = 0.759). The 2.48-fold increased risk of hypertension in African Americans was greater than the 1.85-fold increased risk in Mexican Americans and non-Hispanic Whites (*p* = 0.017).

**Table 3:** Risk of hypertension as a function of hyperuricemia.

Sample	Sample Size	Odds Ratio (95 % Confidence Interval)	*P*
HUFS	1,006	2.90 (2.04, 4.13)	3.09 × 10^−9^
NHANES Mexican American	2,743	1.78 (1.32, 2.39)	1.37 × 10^−4^
NHANES non-Hispanic White	6,674	1.87 (1.61, 2.18)	5.51 × 10^−16^
NHANES non-Hispanic Black	2,761	2.30 (1.81, 2.92)	8.46 × 10^−12^

Hypertension was defined as being diagnosed by a doctor as having high blood pressure or hypertension, taking anti-hypertensive medication, systolic blood pressure ≥ 140 mm Hg, or diastolic blood pressure ≥ 90 mm Hg. The risk of hypertension as a function of hyperuricemia was assessed using logistic regression, adjusted for age, sex, and body mass index (and individual admixture proportion in the HUFS).

### Global distribution of rs2855126

**Fig. 7:**
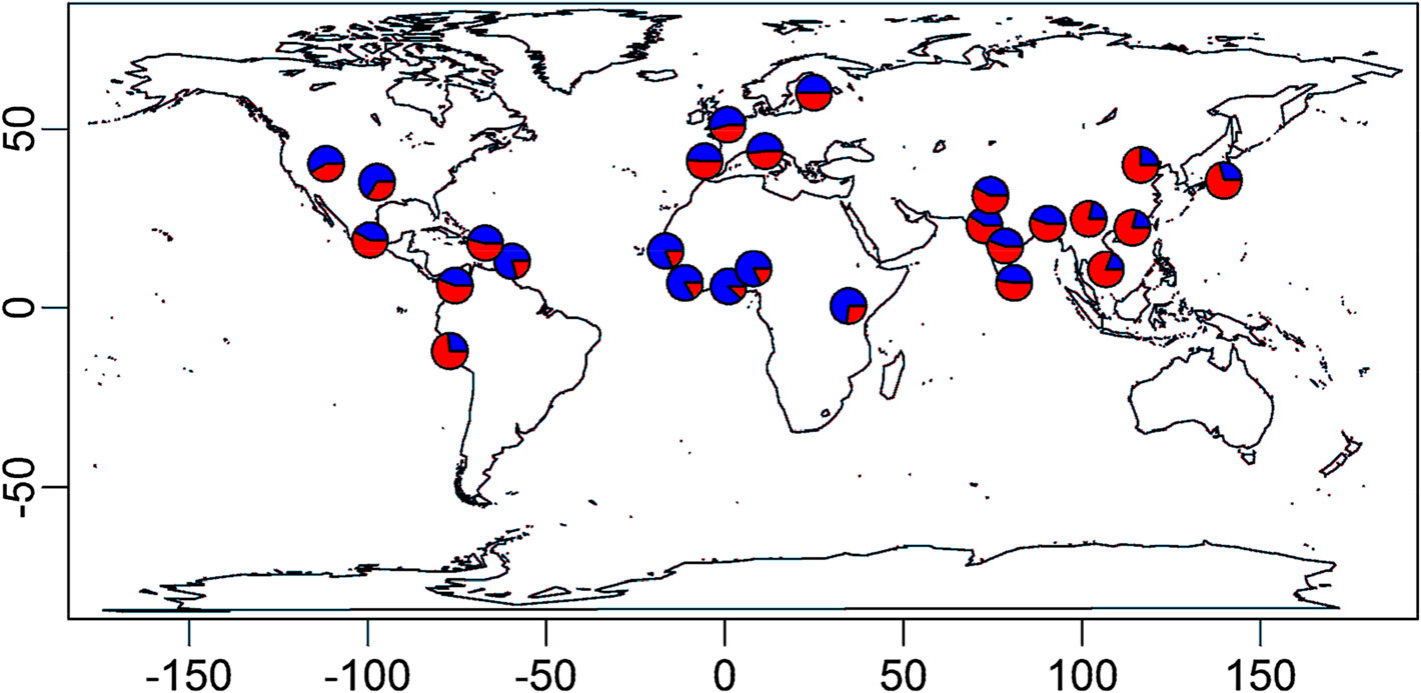
Global distribution of rs2855126. Blue represents the ancestral allele and red represents the derived allele.

To place our findings into an evolutionary context, we examined the global distribution of allele frequencies at rs2855126 (http://browser.1000genomes.org). The highest frequencies of the ancestral allele (>80 %) were observed in West Africa and the lowest frequencies (<20 %) were observed in Southeast Asia (Fig. 7). Globally, rs2855126 is highly differentiated (*F*_*ST*_ = 0.165), with the highest pairwise value between Esan in Nigeria and Kinh in Ho Chi Minh City, Vietnam (*F*_*ST*_ = 0.472).

## Discussion

Increased levels of serum uric acid can result from increased production and/or reduced excretion. In African Americans, we found that the β-globin locus is associated with serum uric acid levels, independent of kidney function. Specifically, we demonstrated that the ancestral allele C at rs2855126, located upstream of *HBG1*, is associated with increased serum uric acid levels and higher expression of a reporter gene relative to the derived allele. We also found that hyperuricemia is associated with markers of increased hemolysis, but not anemia, and an increased risk of hypertension.

Association of the β-globin locus with serum uric acid levels has not been reported in meta-analyses of GWAS in individuals of European ancestry [9, 26, 27]. Epistatic interactions have been described among genetic variants associated with thalassemias and sickle cell traits [28]. Furthermore, looping interactions involving the locus control region [29] and coordinated expression of the β-globin locus with the α-globin locus on chromosome 16 [30] raise the possibility that additive models of genetic association based on single markers may be inadequate to capture *in toto* the variance of serum uric acid levels explained by variants in hemoglobin genes.

According to the catalog of published GWAS [31], γ-globin has been associated with disease severity in β^0^-thalassemia/HbE disease and fetal hemoglobin levels [32, 33]. Fetal hemoglobin, consisting of two copies of α-globin and two copies of γ-globin, is protective against malaria by slowing growth of *Plasmodium falciparum* in erythrocytes [34, 35]. Point mutations resulting in increased fetal hemoglobin levels by increasing γ-globin gene expression also reduce the severity of hemoglobinopathies [23, 36]. We hypothesize that the alleles associated with increased levels of serum uric acid are associated with up-regulation of γ-globin gene expression. The ancestral allele at rs2855126 ranges in frequency from 88 % in the ESN (Esan in Nigeria) sample to 19 % in the KHV (Kinh in Ho Chi Minh City, Vietnam) sample [20], indicating a very high level of genetic differentiation at this locus, consistent with natural selection favoring the ancestral allele in West Africa but favoring the derived allele in Southeast Asia. *P. falciparum* accounts for most malaria in Africa whereas *P. vivax* accounts for most malaria in Asia and South America and is rare in Africa [37]. Whereas *P. falciparum* can infect erythrocytes of all ages, *P. vivax* targets reticulocytes and is less lethal [37], consistent with stronger pressure for the protective effect of the ancestral allele in response to *P. falciparum* infection.

The malarial parasite *Plasmodium falciparum* requires hypoxanthine (interconverted into uric acid by xanthine dehydrogenase) because it cannot synthesize purines *de novo* [38]. Uric acid is released when infected erythrocytes rupture. Uric acid has potent anti-oxidant properties and is the most abundant anti-oxidant in plasma [1, 39], but it also can act as a pro-oxidant. As a pro-oxidant, uric acid alters intracellular redox and activates ERK1/2 and p38 MAPK signaling pathways [40]. Consequently, uric acid induces TNF, IL-6, IL-1β, and IL-10, which can clear the parasite but can also damage the host [41, 42].

Five lines of evidence support the hypothesis that rs2855126 is part of a regulatory element. From the ENCODE data, (1) enhancer histone marks in K562 cells indicate a strong enhancer state, and (2) ChIP-seq data indicate the presence in K562 cells of a binding site for POL2A [43]. (3) RNA protection assays/reverse transcriptase-PCR transcription analyses indicate the presence of a major transcription start site [44]. (4) There is evidence of cis-eQTL activity at rs2855126 for *HBG1* and *HBG2* in monocytes [45]. Finally, (5) we found allele-specific activity in reporter gene expression in transient transfections of K562 and 293 T cells.

Gene expression levels are heritable and several diseases have been associated with non-coding SNPs [46, 47]. Here, we demonstrated that an intergenic region in the β-globin gene cluster acts as an enhancer. We hypothesize that up-regulation of γ-globin leads to hemoglobin imbalance. Hemoglobin associated with the red blood cell membrane induces oxidative damage to specific cytoskeletal components [48], and oxidative stress plays a major role in hemolysis [49, 50]. The degree of imbalance between α- and non-α-globin chain synthesis and the size of the free α-chain pool are also implicated in the severity and clinical manifestations of β-thalassemia [51].

Hyperuricemia is a risk factor for hypertension [52]. Uric acid rapidly and irreversibly inactivates nitric oxide [53]. Cell-free hemoglobin also limits nitric oxide bioavailability [54, 55]. Inhibition of nitric oxide in blood vessels leads to reduced elasticity and increased blood pressure [56]. Elevated serum uric acid levels also induce activation of the renin-angiotensin pathway, leading to vasoconstriction and acute elevation of blood pressure [57]. This acute elevation of blood pressure is reversible by reducing serum uric acid levels or blocking the renin-angiotensin pathway [57]. Uric acid uptake into vascular smooth muscle cells leads to arteriolosclerosis and impaired pressure natriuresis, leading to chronic elevation of blood pressure that is uric acid-independent and sodium-sensitive [57].

## Conclusions

We discovered an association of the β-globin locus with serum uric acid levels in admixed African Americans. At rs2855126, the ancestral allele is associated with higher levels of serum uric acid and higher levels of reporter gene expression. We hypothesize that enhancer activity associated with the ancestral allele drives higher expression of γ-globin, leading to increased levels of fetal hemoglobin and conferring protection against malaria independent of hemoglobin S. We also hypothesize that higher expression of γ-globin leads to hemoglobin imbalance, in turn leading to increased hemolysis and higher levels of serum uric acid. Subsequently, higher levels of serum uric acid are associated with increased risk of hyperuricemia and hypertension.

## Additional files

Additional file 1:
**Primers used for cloning of each SNP fragment.** (DOCX 20 kb) Additional file 2:
**Annotation of SNPs associated with serum uric acid levels.** (XLSX 14 kb) Additional file 3:
**List of predicted transcription factor binding sites using MatInspector and RegulomeDB.** (DOC 48 kb)
