# Supplementary material for: Evolutionary context for the association of γ-globin, serum uric acid, and hypertension in African Americans
Source: BMC Med Genet. 2015 Nov 5;16:103. doi: 10.1186/s12881-015-0249-z (PMC4684912; doi:10.1186/s12881-015-0249-z)
Supplement: Additional file 1: — Primers used for cloning of each SNP fragment. (DOCX 20 kb) [file 12881_2015_249_MOESM1_ESM.docx]

**Supplementary Table 1.** Primers used for cloning of each SNP fragment. Restriction enzyme recognition sites are underlined. All primers sequences are given in the 5′→3′ orientation.

| SNP | Primer sequences (F=Forward, R=Reverse) | PCR product size (bp) |
| --- | --- | --- |
| rs2855125  MluI-BglII | F: ACGCGTTTTTCTCAGCCCTTGTGAGG  R: AGATCTTTTTCTGCTGGTTCCTGGTC | 699 |
| rs2855126  MluI-BglII | F: ACGCGTACATGCTGAATCCCCAACTC  R: AGATCTGTAGGAAGGGCATGTGGAAA | 598 |
| rs11036415  MluI-SmaI | F: ACGCGTCAGCCAAAAGGACGCACTAT  R: CCCGGGTTCAGGTCACAGAGGTAGCAGA | 526 |
| rs11036496  MluI-SmaI | F: ACGCGTCCTTTGTCTTCAGCTACCA  R: CCCGGGGTGCAGACTCCACTGGCA | 403 |
| rs4320977  MluI-SmaI | F: ACGCGTGGTGGCTCAGGCTTGTAAAC  R: CCCGGGGAATGTGTTTGTGAGGGAGGA | 536 |
| rs4348933  MluI-BglII | F: ACGCGTGAATCTATCTGGTCCTGGA  R: AGATCTGAGGACAGACAGACAATA | 496 |
