# Supplementary material for: Evolutionary context for the association of γ-globin, serum uric acid, and hypertension in African Americans
Source: BMC Med Genet. 2015 Nov 5;16:103. doi: 10.1186/s12881-015-0249-z (PMC4684912; doi:10.1186/s12881-015-0249-z)
Supplement: Additional file 3: — List of predicted transcription factor binding sites using MatInspector and RegulomeDB. (DOC 48 kb) [file 12881_2015_249_MOESM3_ESM.doc]

**Additional File 3.** List of predicted transcription factor binding sites using MatInspector and RegulomeDB.

| **SNP** | **MatInspector** | | | **RegulomeDB** |
| --- | --- | --- | --- | --- |
|  | **Predicted transcription factors** | **Matrix similarity** | **Sequences**  (S indicates each SNP position and capital letters indicate the transcription factor binding site) |  |
| rs2855126 | | MEF2 | | --- | | NKX3.1 | | PHOX2 | | | 0.775 | | --- | | 0.859 | | 0.902 | | | gaaagagaAAAAaataagcttc**s** | | --- | | **s**gtgttCAGTggattagaa | | gtttcTAATccactgaacac**s** | | POL2 |
| rs11036496 | | MARE | | --- | | TCF11 | | SOX30 | | SOX5 | | | 0.926 | | --- | | 0.907 | | 0.924 | | 0.906 | | | ct**s**tGCTGtgtcatgcaggtc | | --- | | cctgcaTGACacagca**s**aggc | | ctaggACAAttatggctgcct**s**tgc | | ctaggACAAttatggctgcct**s**tgc | | NRF2  TCF11 |
| rs4348933 | | EN1 | | --- | | PCE1 | | ATBF1 | | | 0.795 | | --- | | 0.901 | | 0.808 | | | taa**s**tgagTTTAtttgcac | | --- | | gtttcTAA**S**tgagttta | | ttttgtttctAA**S**Tgag | | FOXP1 |
